# COVID-19 in children: an approach for multisystem inflammatory syndrome

**DOI:** 10.1186/s43054-021-00082-y

**Published:** 2021-10-26

**Authors:** Haneen K. Morsy, Noha S. Tohamy, Hager M. Abd El Ghaffar, Rana Sayed, Nagwa A. Sabri

**Affiliations:** Clinical Pharmacy Department, Faculty of Pharmacy, Ain Shams University, Cairo, Egypt

**Keywords:** Multisystem inflammatory syndrome, Coronavirus, Pediatrics, Kawasaki, Immunoglobulins

## Abstract

**Background and objectives:**

Children suffering from coronavirus disease (COVID-19) usually present with mild symptoms and show lower mortality rates than adults. However, there have been several recent reports of more severe hyperinflammatory presentation in pediatric COVID-19 patients. This review article aims to summarize the current literature available on the main clinical features and management approaches of multisystem inflammatory syndrome in children (MIS-C).

**Methods:**

The authors searched different indexing databases for observational and interventional studies using search terms including “Coronavirus, COVID-19, pediatric, MIS-C, Kawasaki, and inflammation.” The retrieved publications were further assessed for relevance to the topic. Only relevant articles were included in writing this review article.

**Main body:**

Multisystem inflammatory syndrome in children (MIS-C) is a hyperinflammatory syndrome temporally related to severe acute respiratory syndrome coronavirus-2 (SARS-CoV-2) infection in pediatrics. It is characterized by persistent fever, rash, elevated inflammatory markers, and multiorgan failure with increasing rates of cardiovascular and gastrointestinal involvement. The exact pathophysiologic mechanisms of MIS-C are still unknown, but it is postulated to be due to an exaggerated immune response to SARS-CoV-2 infection. Multisystem inflammatory syndrome in children is diagnosed by exclusion of other underlying causes of organ failure. There is a lack of clinical evidence on the management of MIS-C. The current guidelines depend mainly on expert opinion based on the management of other hyper-inflammatory syndromes in children. Patients suffering from MIS-C are treated with intravenous immunoglobulin (IVIg), corticosteroids, infliximab, tocilizumab, and anakinra.

**Conclusions:**

Despite the growing reports on COVID-19 in children, there is still a lot to elucidate on the pathophysiology, diagnosis, and subsequent management of MIS-C. Further trials are needed to investigate new approaches to manage MIS-C. Specific evidence-based guideline for management of MIS-C should be tailored to the current available information on MIS-C.

## Background

Cases of coronavirus disease (COVID-19) were first reported in later 2019 in China. Coronavirus disease (COVID-19) is caused by severe acute respiratory syndrome coronavirus-2 (SARS-CoV-2), a member of beta-coronavirus family [1]. In March 2020, the World Health Organization (WHO) declared COVID-19 a pandemic. Infection by SARS-CoV-2 is mostly asymptomatic. Some patients may develop mild-to-moderate respiratory symptoms. A minority of patients develop severe disease. The risk of severe COVID-19 increases in elderly, immune compromised patients, and those suffering from chronic comorbidities. Previous reports of SARS-CoV-2 infection indicated that young children were spared from severe infection [2, 3].

The main mode of transmission of SARS-CoV-2 infection is via respiratory droplets [4]. Early reports during the pandemic suggested children have milder symptoms during acute infection. Pediatric COVID-19 typically presents with mild symptoms such as cough, fever, sore throat, and diarrhea. Children present with less frequent lower respiratory tract symptoms and show lower mortality rates as compared to adults [5]. Although COVID-19 in children is usually mild, a rare novel post-COVID syndrome known as multisystem inflammatory syndrome in children (MIS-C) has been reported recently in children and adolescents. MIS-C was reported to be similar to other hyper-inflammatory syndromes in children such as Kawasaki disease shock syndrome and toxic shock syndrome. The main differences between Kawasaki disease and MIS-C are summarized in Table 1 [1, 6].

**Table 1:** The major differences between Kawasaki disease and multisystem inflammatory syndrome in children

Characteristics	Kawasaki disease	Multisystem inflammatory syndrome in children
**Age of presentation**	Less than 5 years	Children aged 8–10 years
**Gender**	Male > female	Male > female
**Fever**	Present	Present
**Cutaneous sign**	Seen in most patients	Seen in < 50% of patients
**Lymphadenopathy**	More common	Not common
**Hemodynamic instability and ICU support**	Less than 5% of patients develop shock syndrome	Present in almost all patients
**Cardiovascular complication**	Symptomatic myocarditis is not common	Cardiac dysfunction is seen at presentation; severe myocarditis and pericarditis are more common
**Predominant symptoms**	Gastrointestinal symptoms are not prominent	Gastrointestinal manifestations (abdominal pain, diarrhea) present in > 80% patients
**Inflammatory markers**	Neutrophilic leukocytosis is usual	Lymphopenia is common; cytokine storm is more severe; extremely high levels of N-terminal brain natriuretic peptide, Troponins and D-dimer
**Organ dysfunction**	Multiorgan dysfunction is not common	Multiorgan dysfunction seen
**Etiology**	No identifiable cause	Post-infectious syndrome. SARS-CoV-2 serology is usually positive; in seronegative patients, there is usually history of contact with an individual having COVID-19 infection
**Management**	Intravenous immunoglobulin; steroid; interleukin-1 blockers	Intravenous immunoglobulin; steroids; interleukin-1 blockers; interleukin-6 inhibitors

*SARS-CoV-*2 severe acute respiratory syndrome coronavirus type 2, *COVID-19* coronavirus disease

Multisystem inflammatory syndrome in children (MIS-C) is defined as a clinically severe illness that needs hospitalization with fever, elevated inflammatory markers, and multisystem organ dysfunction in patients with recently proven or probable SARS–CoV-2 infection, and in the absence of an alternative underlying cause [7]. The pathogenesis of MIS-C is unknown, and a post-infectious etiology has not been proven. SARS–CoV-2 antibodies are detectable in the second week after infection. A recent study reported that patients with MIS-C show decreased neutralizing antibody activity against SARS–CoV-2, compared with adults with COVID-related acute respiratory distress syndrome (ARDS) and adults with mild disease [8]. The virus is generally not detected in the respiratory tract of patients with MIS-C. There is not enough data on presence of SARS–CoV2–specific T cells in peripheral blood of MIS-C patients, and the biological role of SARS–CoV-2–reactive T cells, whether protective or even detrimental, is still unclear [9, 10].

According to the centers of disease control (CDC), the diagnostic criteria of MIS-C are children aged less than 21 years with the following clinical criteria: minimum 24-h of fever of at least 38 °C and severe illness requiring hospital admission and with more than 2 organ-system involvement. At least one laboratory evidence of inflammation should be present including an elevated C-reactive protein, erythrocyte sedimentation rate, fibrinogen, procalcitonin, D-dimer, ferritin, lactate dehydrogenase, or interleukin-6; elevated neutrophils; or reduced lymphocytes or decreased albumin. There should be laboratory or epidemiologic evidence of SARS-CoV-2 infection in the absence of an alternative diagnosis [7].

Management of mild COVID-19 in children without symptoms of dyspnea or impaired feeding includes home isolation with use of paracetamol for supportive control of fever, proper hydration and antibiotic therapy for patients showing signs of secondary bacterial infection. Severe cases require intensive care admission to maintain oxygen saturation greater than 92% measured by pulse oximetry and to maintain hemodynamic stability. Severe cases with suspected sepsis need empirical antibiotic therapy within an hour of admission [11].

Remdesivir is approved for management of COVID-19 children aged ≥ 12 years and weighing ≥ 40 kg with a loading intravenous dose of 200 mg infused over 30–120 min followed by 100 mg intravenous every day. Remdesivir is also available for younger children (and those weighing < 40 kg and > 3.5 kg) through an FDA Emergency Use Authorization [11]. Alternative antiviral therapy includes lopinavir/ritonavir combination at a dose of 10 mg/2.5 mg per kg twice daily for maximum 14 days; maximum dose is 400 mg/100 mg twice daily [12]. Hydroxychloroquine showed anti-SARSCoV-2 activity in in vitro studies. It is indicated in confirmed or suspected COVID-19 with severe pneumonia or critically ill patients at a dose of 7–8 mg/kg twice daily for day 1 and then days 2–5, 7–8 mg/kg once a day [13].

The American College of Rheumatology (ACR) guidelines for treatment of MIS-C recommends the use of intravenous immunoglobulin (IVIg) and/or high-dose corticosteroids as first-line therapy in MIS-C patients. Approximately 30–80% patients require adjunctive immunomodulatory therapy to control inflammation. Multiple intravenous pulse methylprednisolone (10–30 mg/kg/day for 3–7 days followed by gradual tapering of oral prednisolone) is beneficial. Other therapeutic options include second dose of IVIg, infliximab, anakinra, and tocilizumab. The ACR guidelines recommended the use of anakinra as a steroid-sparing agent for children with contraindications to long term corticosteroid regimens. However, no recommendations regarding the use of other steroids-sparing agents were made [14].

The number of reported MIS-C cases is increasing. The clinical definition and diagnostic criteria of MIS-C are preliminary. Management of MIS-C is derived empirically from management of similar hyperinflammatory disorders. The treatment outcomes are unclear in this setting especially with increased rates of cardiovascular involvement. This review is constructed to demonstrate the current evidence available on the clinical presentation, laboratory findings, management, and clinical outcomes of MIS-C in literature.

## Methods

The authors searched several indexing scientific databases including Clarivate, PubMed, and Embase for both observational and interventional studies involving MIS-C patients. The retrieved articles were assessed for relevance to the aim of this review article. Relevant articles were thoroughly read and summarized.

## Results

### Association between COVID-19 in children and MIS-C

Several studies have demonstrated the temporal relationship between development of MIS-C and previous SARS-CoV-2 infection. Verdoni et al. divided the patients presenting with Kawasaki-like symptoms to the pediatrics’ department in a single hospital in Italy over a period of 5 years starting January 2015 into 2 groups based on the onset of symptoms either before or after the SARS-CoV-2 outbreak. Eighty percent of those who developed Kawasaki-like symptoms after COVID-19 outbreak tested positive for SARS-CoV-2 IgG, IgM, or both [15]. In the UK, 29 patients were admitted to two hospitals between March and June 2020. Patients’ link with COVID-19 was established in 24 cases. The increase in incidence of MIS-C was lagging by 4 weeks after the first surge of COVID-19 [16].

Similarly, an increase in incidence of Kawasaki- like symptoms in children presenting to the pediatrics’ department in a university hospital in Paris was reported (17 cases in 11 days after COVID-19 outbreak versus 1 case per 2-week period over 2018-2019). Eighty-two percent of those presenting with Kawasaki-like symptoms since April 2020 suffered from SARS-CoV-2 confirmed infection [17]. In India, 19 out of 21 MIS-C patients showed positive SARS-CoV-2 current or previous infection using either polymerase chain reaction or SARS-CoV-2 antibody detection tests. The other two patients came from containment zones of COVID-19 [18].

Diorio et al. studied the main hematologic and immunologic parameters that distinguish between severe COVID-19 and MIS-C in a cohort of 20 patients. Higher TNF-α and IL-10 discriminated between patients with MIS-C and severe COVID-19. The presence of burr cells on blood smears differentiated between patients with severe COVID-19 and those with MIS-C [19].

### Clinical characteristics, demographic features, and laboratory findings of MIS-C

Several studies focused on the differences in patients’ characteristics and clinical presentation between those suffering from Kawasaki disease and MIS-C patients [15, 17, 20]. In Italy, patients who developed Kawasaki-like symptoms after the COVID-19 outbreak had higher incidence, more cardiac involvement, higher need for steroid treatment, and were older compared to those who developed Kawasaki-like symptoms before February 18, 2020 [15]. Toubiana et al. reported that cases presenting with Kawasaki-like symptoms after the COVID-19 outbreak in Paris showed higher incidence of gastrointestinal involvement and increased risk of developing Kawasaki disease shock syndrome. Moreover, African ancestry was associated with increased incidence of Kawasaki- like symptoms after COVID-19. Patients responded favorably to IVIg administration alone or in combination with corticosteroids [17].

Kaushik et al. studied the clinical characteristics and outcomes of SARS-CoV-2-associated MIS-C retrospectively in 33 children. Data was collected from 3 different hospitals in New York. The cohort studied had a median age of 10 years. The majority of children with MIS-C were males. Forty-five percent of the patients had other comorbidities. Patients frequently presented with fever, nausea, vomiting, abdominal pain, and hypotension. Sixty-three percent of patients suffered from a decreased left ventricular ejection fraction. All patients had elevated inflammatory markers including C-reactive protein, D-dimer, procalcitonin, and pro-B-type N-natriuretic peptide [21].

A survey was conducted to assess the differences in MIS-C patients’ evaluation and management in 40 different US hospitals. All hospitals required fever and organ involvement as parts of MIS-C diagnosis. Previous contact with SARS-CoV-2 patient during the 4 weeks preceding symptoms onset was enough for associating MIS-C symptoms to COVID-19. During patient evaluation, polymerase chain reaction testing of nasopharyngeal swabs was done for all patients to confirm SARS-CoV-2 infection. Routine laboratory testing included complete blood picture, liver function tests, kidney function tests, metabolic panel, C-reactive protein, and erythrocyte sedimentation rate. Tests to check for inflammatory markers’ levels, cardiovascular involvement, and coagulopathy were performed in select patients. Exclusion of other infectious diseases was routinely recommended [22].

A multicenter cohort study of 5 Latin American countries (Mexico, Colombia, Brazil, Costa-Rica, and Peru) included 409 confirmed cases of pediatric SARS-CoV-2 of whom 95 cases fulfilled the CDC criteria for diagnosis of MIS-C. The median age of patients was 3 years. Fifty-four percent of the cohort were males. Forty-six percent of patients needed hospital admission. Determinants of need for pediatric intensive care admission include low socioeconomic status, lower respiratory tract infection, gastrointestinal involvement, pre-existing comorbidities, and immunodeficiency [23].

An observational case series in a tertiary hospital in India described the clinical and demographic characteristics of 21 MIS-C patients presenting to the pediatric intensive care unit. Patients presented at a median age of 7 years. Eleven patients were females. Patients complained from fever mainly followed by gastrointestinal symptoms, respiratory distress, and macular rash. Eighty percent of patients suffered from lymphocytopenia. Inflammatory markers were elevated in all patients including C-reactive protein and serum ferritin together with elevated D-dimer levels [18].

In the UK, a cohort of 29 patient showed that two thirds of admitted cases were males. Children of black, Asian, and other minority ethnic background were at higher risk of developing MIS-C compared to Caucasians. The median age of MIS-C patients was 6 years. The chief complaint in all patients was fever. Gastrointestinal and dermatologic involvement was common. Twenty-three patients suffered from lymphopenia. In addition to elevated C-reactive protein, serum ferritin, and D-dimer, some patients had elevated triacylglycerol levels and transaminases. The majority of children showed cardiac involvement [16].

A retrospective cohort was performed in a single tertiary hospital in the UK to assess the clinical findings in children referred to cardiology consultation with confirmed MIS-C. Fifteen patients over a period of 1 month with a median age of 8.8 years met MIS-C diagnostic criteria. The majority of patients were males and all of them were of black, Asian, and other minority ethnic background. All patients suffered from pyrexia and more than 80% reported gastrointestinal involvement. Patients had significantly elevated inflammatory markers (ferritin, erythrocyte sedimentation rate, and C-reactive protein) and cardiac markers (troponin I, creatine kinase, and pro-B-natriuretic peptide). Sixty percent of the patients had echocardiographic abnormalities [24].

Saleh et al. conducted an observational cohort of 398 Egyptian children suffering from confirmed COVID-19. Four patients met the diagnostic criteria of MIS-C. In addition to severe respiratory symptoms, the four patients suffered from bilateral conjunctivitis and maculopapular rash. They had elevated liver transaminases, ferritin, and lactate dehydrogenase levels. Moreover, two patients showed coronary dilatation and left ventricular dysfunction in echocardiographic studies [25].

A single-center study showed that 5 (4 males and 1 female) of 88 children suffering from COVID-19 met the diagnostic criteria of MIS-C. The patients’ age ranged between 8 and 11 years. They had normal echocardiographic findings but complained of abdominal pain, dehydration, and rash [26]. Mahmoud et al. described a group of 64 MIS-C patients. Eighty-six percent of patients presented with cardiac abnormalities while 78% showed gastrointestinal involvement [27].

A retrospective cohort of 44 MIS-C patients investigated the clinical and biochemical features of liver involvement and their correlation to disease severity. Hepatitis was reported to be related to more severe manifestations of MIS-C. Liver involvement was associated with higher rates of shock, longer hospitalization, and higher need for ventilatory support. Patients with hepatitis had significantly higher inflammatory markers [28].

### Management and clinical outcomes

The current management recommendations for MIS-C are based on treatment guidelines of similar hyperinflammatory diseases such as Kawasaki disease and toxic shock syndrome. Dove et al. described the management approach of MIS-C patients in 40 US hospitals. The most commonly used treatment option was IVIg regardless of disease severity. A second dose of IVIg was recommended in cases refractory to the first dose. Aspirin was used for mild to moderate cases while corticosteroids were saved for moderate or severe symptoms. Anticoagulation using unfractionated or low molecular weight heparin was applied to severe cases. Anakinra and vasopressors were used for severe cases. The majority of hospitals recommended follow-up similar to Kawasaki disease. Speciality follow-up visits included cardiology, rheumatology, infectious diseases, and hematology [22]. In 3 New York hospitals, patients were treated using IVIg, corticosteroids, tocilizumab, and remdesivir with favorable clinical response. Patients required a median pediatric intensive care unit stay of 4.7 days and a hospital stay of 7.8 days [21].

A cohort in an Indian hospital reported that most patients needed respiratory support and vasopressors. Corticosteroids were the first line therapy in this hospital followed by IVIg due to cost issues. Tocilizumab was saved for severe cases that did not respond to steroids and IVIg. Two patients (out of 21) died due to MIS-C associated cardiac dysfunction. Five patients needed transient insulin treatment while 2 patients needed high glucose infusion for hypoglycemia [18]. In a retrospective cohort of 44 children, liver involvement was associated with more severe presentation and worse clinical outcome. One patient suffered from liver failure and needed vasopressors, dialysis, and mechanical ventilation to manage subsequent multiorgan failure [28].

Felsenstein et al. described management of 29 MIS-C patients with a single dose or 2 doses of IVIg alone or in combination with methylprednisolone followed by short prednisolone taper. One patient needed addition of anakinra and one patient needed addition of infliximab to IVIg and steroids combination. All patients clinically improved. Patients were followed-up for 2 weeks after presentation. Forty-one percent of patients had persistent symptoms on the 2-week follow-up [16].

Fifteen children with confirmed MIS-C and cardiac involvement were treated primarily using IVIg. Five patients received intravenous methylprednisolone. None of the patients needed anticoagulation nor aspirin. The median duration of hospitalization was 12 days. More than 80% of the patients showed stable clinical and echocardiographic findings on follow-up [24]. Shahin et al. reported full recovery of 5 MIS-C patients upon management using antiplatelets, IVIg, and pulse steroid therapy [26]. On the other hand, Mahmoud et al. reported 9% mortality among MIS-C in-patients treated with IVIg and methylprednisolone (100% and 94%, respectively) [27].

### Hypercoagulability and thromboembolic events

Novel COVID-19 patients generally have a higher risk of thromboembolic complications. Del Borrello et al. shared their experience in managing hypercoagulability in a prospective cohort of 35 hospitalized SARS-CoV-2 pediatric patients. Patients were divided into 2 groups: a group of 30 children were admitted due to SARS-CoV-2 infection but were not meeting MIS-C diagnostic criteria, and a group of 6 children were diagnosed as MIS-C patients. D-dimer levels were consistently elevated in COVID-19 patients regardless of disease severity. D-dimer levels changed over the course of the disease with slight elevation during periods of symptoms worsening. Patients meeting MIS-C diagnostic criteria had a significantly higher median D-dimer level compared to COVID-19 patients (1906 versus 817 ng/mL, respectively). Moreover, a D-dimer level greater than 1000 ng/mL was an appropriate cutoff value to differentiate between MIS-C patients and COVID-19 cases with a 100% sensitivity and 83.3% specificity. Prophylactic anticoagulation was initiated in 6 patients and continued till patient discharge or resolution of thromboembolic risk, whichever achieved first. Four patients received enoxaparin while 2 patients received unfractionated heparin. None of the patients developed bleeding nor thromboembolic complications [29].

## Discussion

Several studies have demonstrated the temporal relationship between development of MIS-C and previous SARS-CoV-2 infection [15–18]. The time-lag between COVID-19 diagnosis and development of MIS-C and the delay of the surge of MIS-C cases after the community peak in COVID-19 incidence support the hypothesis that MIS-C is a heightened inflammatory response to SARS-CoV-2 infection. Patients suffering from MIS-C had considerably higher TNF-α and IL-10 levels compared to severe COVID-19 patients [19]. The higher TNF-α level in MIS-C patients could be an attractive for future investigation of the use of infliximab as an anti-TNF agent for the management of MIS-C.

Studies focusing on the main differences in demographics and clinical manifestation between patients with Kawasaki disease and MIS-C patients showed that patients suffering from MIS-C were older with males being at higher risk of developing MIS-C than females. Patients with MIS-C presented with fever and had higher incidence of cardiovascular and gastrointestinal involvement [15, 17, 18, 20, 21].

The exact reason for higher risk of development of severe post-COVID-19 complications in males compared to females is still unknown. One of the proposed potential reasons is the difference in expression of angiotensin-converting enzyme-2 (ACE-2) receptor which plays a protective role against COVID-19. Males showed lower expression of ACE-2 receptor. Female sex hormone (estrogen) is implicated in higher expression of cardioprotective ACE-2 receptor [30]. Similarly, the older age of children suffering from MIS-C could be attributed to the difference in ACE-2 expression and the levels of circulating angiotensin [31]. The increased rate of cardiovascular involvement in MIS-C could be attributed to higher levels of circulating interleukin-6 and CRP which were associated with cardiomyopathy in bacterial infections [32]. Moreover, coronary artery dilatation observed in some MIS-C and Kawasaki disease patients could be due to coronary vessels damage caused by neutrophil activation as a response to circulating immune complexes [33].

Patients with MIS-C respond favorably to treatment with IVIg alone or combined with intravenous corticosteroids. Remdesivir is used for patients with active COVID-19 infection. Anti-inflammatory agents such as tocilizumab, infliximab, and anakinra are added in severe and refractory MIS-C cases. In addition, hemodynamic support and oxygen supplementation are provided to select patients when required. Both adult and pediatric patients suffering from novel COVID-19 show higher susceptibility to thromboembolic events. Similarly, patients suffering from MIS-C had significantly elevated D-dimer levels [29].

## Conclusions

Despite the growing reports on COVID-19 in children, there is still a lot to elucidate on the pathophysiology, diagnosis, and subsequent management of MIS-C. Collaboration between pediatricians, pediatric intensivists, cardiologists, infectious disease specialists, and immunologists is essential to address diagnosis and management of MIS-C. Further studies are needed to advance our understanding of the pathophysiology of MIS-C and the mechanisms underlying cardiovascular involvement which would help developing new strategies to manage MIS-C. The hypothesis of being an immune-mediated injury due to SARS-CoV-2 infection necessitates investigating the risk of MIS-C being precipitated by COVID-19 vaccines before initiating mass vaccination in children.

## Data Availability

Not applicable

## Abbreviations

COVID-19: Coronavirus disease
MIS-C: Multisystem inflammatory syndrome in children
SARS-CoV-2: Severe acute respiratory syndrome coronavirus-2
IVIg: Intravenous immunoglobulin
ACE-2: Angiotensin-converting enzyme-2
ACR: American College of Rheumatology

## References

[1] Kabeerdoss J, Pilania RK, Karkhele R, Kumar TS, Danda D, Singh S (2021). Severe COVID-19, multisystem inflammatory syndrome in children, and Kawasaki disease: immunological mechanisms, clinical manifestations and management. Rheumatol Int.

[2] Bialek S, Gierke R, Hughes M, McNamara LA (2020). Coronavirus disease 2019 in children—United States, february 12–april 2, 2020. MMWR Morb Mortal Wkly Rep.

[3] Parri N, Lenge M, Buonsenso D (2020). Children with COVID-19 in pediatric emergency departments in Italy. N Engl J Med.

[4] World Health Organization (2020) Transmission of SARS-CoV-2: implications for infection prevention precautions: scientific brief, 09 July 2020. World Health Organization. https://www.who.int/news-room/commentaries/detail/transmission-of-sars-cov-2-implications-for-infectionprevention-precautions

[5] Qiu H, Wu J, Hong L, Luo Y, Song Q, Chen D (2020). Clinical and epidemiological features of 36 children with coronavirus disease 2019 (COVID-19) in Zhejiang, China: an observational cohort study. Lancet Infect Dis.

[6] Han SB, Lee SY (2020). Macrophage activation syndrome in children with Kawasaki disease: diagnostic and therapeutic approaches. World J Pediatr.

[7] Levin M, Schneider J, Marconi VC, Morris SB, Belay E (2020). Multisystem inflammatory syndrome in children (MIS-C) associated with coronavirus disease 2019 (COVID-19).

[8] Weisberg SP, Connors T, Zhu Y, Baldwin M, Lin WH, Wontakal S et al (2020) Antibody responses to SARS-CoV2 are distinct in children with MIS-C compared to adults with COVID-19. medRxiv. 10.1101/2020.07.12.20151068

[9] Wölfel R, Corman VM, Guggemos W, Seilmaier M, Zange S, Müller MA (2020). Virological assessment of hospitalized patients with COVID-2019. Nature.

[10] Sette A, Crotty S (2020). Pre-existing immunity to SARS-CoV-2: the knowns and unknowns. Nat Rev Immunol.

[11] WHO (2020). Clinical management of severe acute respiratory infection when novel coronavirus (nCoV) infection is suspected, Interim guidance, 13 March 2020.

[12] Cao B, Wang Y, Wen D, Liu W, Wang J, Fan G (2020). A trial of lopinavir-ritonavir in adults hospitalized with severe COVID-19. N Engl J Med.

[13] Liu J, Cao R, Xu M, Wang X, Zhang H, Hu H (2020). Hydroxychloroquine, a less toxic derivative of chloroquine, is effective in inhibiting SARS-CoV-2 infection in vitro. Cell Discov.

[14] Henderson LA, Canna SW, Friedman KG, Gorelik M, Lapidus SK, Bassiri H (2020). American College of Rheumatology clinical guidance for pediatric patients with multisystem inflammatory syndrome in children (MIS-C) associated with SARS-CoV-2 and hyperinflammation in COVID-19. Version 2.

[15] Verdoni L, Mazza A, Gervasoni A, Martelli L, Ruggeri M, Ciuffreda M (2020). An outbreak of severe Kawasaki-like disease at the Italian epicentre of the SARS-CoV-2 epidemic: an observational cohort study. Lancet (London, England).

[16] Felsenstein S, Willis E, Lythgoe H, McCann L, Cleary A, Mahmood K (2020). Presentation, treatment response and short-term outcomes in paediatric multisystem inflammatory syndrome temporally associated with SARS-CoV-2 (PIMS-TS). J Clin Med.

[17] Toubiana J, Poirault C, Corsia A, Bajolle F, Fourgeaud J, Angoulvant F (2020). Kawasaki-like multisystem inflammatory syndrome in children during the covid-19 pandemic in Paris, France: prospective observational study. BMJ (Clinical research ed).

[18] Shobhavat L, Solomon R, Rao S, Bhagat I, Prabhu S, Prabhu S (2020). Multisystem inflammatory syndrome in children: clinical features and management-intensive care experience from a pediatric public hospital in Western India. Indian J Crit Care Med.

[19] Diorio C, Henrickson SE, Vella LA, McNerney KO, Chase J, Burudpakdee C (2020). Multisystem inflammatory syndrome in children and COVID-19 are distinct presentations of SARS-CoV-2. J Clin Invest.

[20] Consiglio CR, Cotugno N, Sardh F, Pou C, Amodio D, Rodriguez L (2020). The immunology of multisystem inflammatory syndrome in children with COVID-19. Cell.

[21] Kaushik S, Aydin SI, Derespina KR, Bansal PB, Kowalsky S, Trachtman R (2020). Multisystem inflammatory syndrome in children associated with severe acute respiratory syndrome coronavirus 2 infection (MIS-C): a multi-institutional study from New York City. J Pediatr.

[22] Dove ML, Jaggi P, Kelleman M, Abuali M, Ang JY, Ballan W (2021). Multisystem inflammatory syndrome in children: survey of protocols for early hospital evaluation and management. J Pediatr.

[23] Antúnez-Montes OY, Escamilla MI, Figueroa-Uribe AF, Arteaga-Menchaca E, Lavariega-Saráchaga M, Salcedo-Lozada P (2021). COVID-19 and multisystem inflammatory syndrome in Latin American children: a multinational study. Pediatr Infect Dis J.

[24] Ramcharan T, Nolan O, Lai CY, Prabhu N, Krishnamurthy R, Richter AG (2020). Paediatric inflammatory multisystem syndrome: temporally associated with SARS-CoV-2 (PIMS-TS): cardiac features, management and short-term outcomes at a UK tertiary paediatric hospital. Pediatr Cardiol.

[25] Saleh NY, Aboelghar HM, Salem SS, Ibrahem RA, Khalil FO, Abdelgawad AS (2021). The severity and atypical presentations of COVID-19 infection in pediatrics. BMC Pediatr.

[26] Shahin W, Rabie W, Alyossof O, Alasiri M, Alfaki M, Mahmoud E (2021). COVID-19 in children ranging from asymptomatic to a multi-system inflammatory disease: a single-center study. Saudi Med J.

[27] Mahmoud S, Fouda EM, Kotby A, Ibrahim HM, Gamal M, El Gendy YG (2021). The “Golden Hours” algorithm for the management of the multisystem inflammatory syndrome in children (MIS-C). Glob Pediatr Health.

[28] Cantor A, Miller J, Zachariah P, DaSilva B, Margolis K, Martinez M (2020). Acute hepatitis is a prominent presentation of the multisystem inflammatory syndrome in children: a single-center report. Hepatology (Baltimore, Md).

[29] Del Borrello G, Giraudo I, Bondone C, Denina M, Garazzino S, Linari C (2021). SARS-COV-2–associated coagulopathy and thromboembolism prophylaxis in children: a single-center observational study. J Thromb Haemost.

[30] Ortona E, Pagano MT, Peruzzu D, Ruggieri A, Gagliardi MC (2020). Opinion: vitamin D and sex differences in COVID-19. Front Endocrinol.

[31] Malviya A, Mishra A (2021). Childhood multisystem inflammatory syndrome: an emerging disease with prominent cardiovascular involvement—a scoping review. SN Compr Clin Med.

[32] Rowley AH (2020). Understanding SARS-CoV-2-related multisystem inflammatory syndrome in children. Nat Rev Immunol.

[33] Menikou S, Langford PR, Levin M (2019). Kawasaki disease: the role of immune complexes revisited. Front Immunol.

